# Acceptance of the Respiratory Syncytial Virus Vaccine in Pregnant Individuals

**DOI:** 10.1097/og9.0000000000000026

**Published:** 2024-08-08

**Authors:** Tonya Tucker, Zoe Garvey, David O'Sullivan, Alexandra Loza

**Affiliations:** University of Connecticut Obstetrics and Gynecology Residency Program and the University of Connecticut School of Medicine, Farmington, and the Department of Maternal Fetal Medicine and the Department of Research Administration, Hartford Healthcare, Hartford, Connecticut.

## Abstract

The respiratory syncytial virus (RSV) vaccine was officially offered to pregnant individuals in 2023–2024, with the purpose of providing immunity to infants and young children. We performed a retrospective review to determine the rate of offering the RSV vaccine and acceptance of the vaccine among patients between 32 0/7 and 36 6/7 weeks of gestation. A prospective survey was administered to examine barriers to RSV vaccination. We identified 206 patients, including 111 (53.8%) who were offered the vaccine and 95 (46.1%) who were not offered the vaccine. Of those who were offered the vaccine, 62 (55.9%) accepted and 49 (44.1%) declined. Individuals who declined had significantly higher rates of declining other vaccines in pregnancy (*P*<.001). More than half of those who declined the RSV vaccine reported both accepting the RSV antibody for their newborn and plans of accepting the vaccine in subsequent pregnancies.

## Abstract 2

Acceptance of other vaccines in pregnancy was strongly associated with acceptance of the respiratory syncytial virus (RSV) vaccine, suggesting overall vaccine hesitancy among pregnant individuals rather than specific hesitancy toward the RSV vaccine.

Pfizer's respiratory syncytial virus (RSV) vaccine, Abrysvo, was introduced in the pregnant population in 2023. The American College of Obstetricians and Gynecologists and the Society for Maternal-Fetal Medicine recommend the RSV vaccine for pregnant individuals between 32 0/7 and 36 6/7 weeks of gestation during RSV season.^1,2^ In addition, the RSV antibody injection, Nirsevimab (Beyfortus), was available for infants if no maternal RSV vaccine was given.^1,3^ This recommendation stems from the MATISSE (Maternal Immunization Study for Safety and Efficacy) phase 3 randomized clinical trial.^4^ Our primary objective was to analyze the acceptance of the RSV vaccine among pregnant individuals in the first season of its offering.

## METHODS

We conducted a retrospective cohort study of patients receiving prenatal care in two clinics during RSV vaccine season (September 2023–January 2024) who were between 32 0/7 and 36 6/7 weeks of gestation. The vaccine was available in the clinics starting on October 23, 2023. Patients with qualifying visits before were excluded. The primary outcome was RSV vaccine acceptance rate. Patients who declined the vaccine were offered participation in a phone survey. Primary outcomes of the survey included qualitative data regarding reasons for declining the vaccine, satisfaction with counseling, acceptance of newborn RSV antibodies, and future feelings regarding the vaccine. Secondary outcomes were acceptance of other vaccines in pregnancy; maternal age; multiparity; race and ethnicity; and pregnancy outcomes inclusive of term delivery, delivery mode, hypertensive disorders of pregnancy, and neonatal outcomes. Race and ethnicity were included to assess cultural perceptions to vaccines. The study was approved by the Hartford HealthCare IRB.

The prospective survey responses were analyzed qualitatively. Retrospective continuous variables were compared between the two groups using Student's *t* test or Mann-Whitney *U* tests. Chi square or Fisher exact tests were used for categorical variables. *P*<.05 was deemed statistically significant. SPSS 29 was used for quantitative analyses; Microsoft Word was used for qualitative analyses.

## RESULTS

The study included 206 patients who were eligible for the RSV vaccine. Of those, 111 (53.8%) were offered the vaccine and 95 (46.1%) were not offered the vaccine. Of those who were offered the vaccine, 62 (55.9%) accepted and 49 (44.1%) declined (Appendix 1, available online at http://links.lww.com/AOG/D786). The temporal distribution of vaccines offered was 2.7% in October, 15.3% in November, 39.6% in December, and 42.3% in January (*P*<.001). The mean gestational age at acceptance was 34 1/7 weeks (±1 2/7 weeks). The median date received was December 28, 2023.

Comparing the groups that accepted and declined the RSV vaccine, one patient (1.62%) compared with 17 (34.7%), respectively, declined the Tdap (tetanus toxoid, reduced diphtheria toxoid, and acellular pertussis) vaccine; 12 (19.4%) compared with 36 (73.5%), respectively, declined the influenza vaccine, and 11 (17.7%) compared with 41 (83.7%), respectively, declined the coronavirus disease 2019 (COVID-19) vaccine (all *P*<.001) (Table 1).

**Table 1.** Demographics and Medical Characteristics of Individuals Who Accepted the Respiratory Syncytial Virus Vaccine and Those Who Declined

Characteristic	RSV Vaccine		*P*
	Accepted (n=62)	Declined (n=49)	
Age (y)	30.18±6.10	28.33±6.29	.12
BMI (kg/m^2^)	29.31±7.16	29.86±6.38	.67
Multiparity	33 (53.2; 41–65)	32 (65.3, 51–76)	.20
Race*			.14
Asian	1 (1.6; 0–7)	0 (0; 0–5)	
Black	9 (14.5; 7–25)	14 (28.6; 17–42)	
White	52 (83.9; 73–91)	35 (71.4; 58–82)	
Ethnicity*			.30
Hispanic	39 (62.9; 50–74)	26 (53.1; 39–66)	
Non-Hispanic	23 (37.1; 26–49)	23 (46.9; 33–61)	
Month of eligible visit			.84
October	2 (3.2; 1–10)	1 (2.0; 0–9)	
November	10 (16.1; 9–27)	7 (14.3; 6–26)	
December	26 (41.9; 30–54)	18 (36.7; 24–51)	
January	24 (38.7; 27–51)	23 (46.9; 34–61)	
Acceptance of other vaccines			
Tdap	61 (98.4; 93–100)	32 (65.3; 51–78)	**<**.001
Influenza	50 (80.6; 70–89)	12 (26.5; 14–38)	**<**.001
COVID-19	51 (82.3; 71–90)	8 (16.3; 8–29)	**<**.001

RSV, respiratory syncytial virus; BMI, body mass index; Tdap, tetanus toxoid, reduced diphtheria toxoid, and acellular pertussis; COVID-19, coronavirus disease 2019.

Data are mean±SD or n (%; 95% CI) unless otherwise specified.

* Race and ethnicity were self-reported in patient medical records.

The survey had 49 eligible participants, with 35 respondents (71.4%). Reasons for not accepting the RSV vaccine were distrust of new vaccines (80.0%), fear of effects on the fetus (45.7%), not enough time to decide (34.2%), desiring long-term studies (22.8%), and overall vaccine fatigue in pregnancy (17.1%). Of the 35 respondents, 33 (94.2%) agreed that their clinician adequately discussed the vaccine. Twenty-two (62.9%) respondents reported accepting newborn RSV antibody. Twenty-three (65.7%) endorsed acceptance of the vaccine in a future pregnancy. Additional opinions included desiring more time to receive the vaccine (37.1%) and reports of not fully understanding the protection for the baby (28.6%) (Table 2).

**Table 2.** Phone Survey Responses of 35 Individuals Who Declined the Respiratory Syncytial Virus Vaccine

Survey Question	Response	n (%; 95% CI)
Why did you decline the RSV vaccine?	General distrust of new vaccine	28 (80.0; 65–91)
	Fear of harmful effects on the fetus	16 (45.7; 30–62)
	Not enough time to decide; too few visits	12 (34.2; 20–51)
	Desired long-term safety information on vaccine in pregnancy	8 (22.8; 11–39)
	Vaccine fatigue in pregnancy	6 (17.1; 8–32)
Do you feel your clinician discussed the RSV vaccine enough with you?	Yes	33 (94.2; 83–99)
Did you accept the RSV antibody for your newborn?	Yes	22 (62.9; 46–77)
Would you accept the RSV vaccine in future pregnancies?	Yes	23 (65.7; 49–80)
What are your future outlooks regarding the RSV vaccine?	Did not fully understand the positive protection the vaccine provided the baby	10 (28.6; 16–45)
	I would like more visits to be able to discuss and decide on accepting the vaccine	13 (37.1; 23–54)
	I would like paper handout on the vaccine by the clinician	6 (17.1; 8–32)

RSV, respiratory syncytial virus.

There were no differences in maternal or neonatal outcomes in our study (Appendix 2, available online at http://links.lww.com/AOG/D786), although the study was not powered for these secondary outcomes.

## DISCUSSION

More than half of pregnant individuals accepted the RSV vaccine in our study. Acceptance of other vaccines in pregnancy was strongly associated with acceptance of the RSV vaccine, suggesting overall vaccine hesitancy among pregnant individuals rather than specific hesitancy toward the RSV vaccine.^5^

Individuals who declined cited both fear of a new vaccine and of effects on the fetus, although 62.9% accepted the new RSV antibody for their newborns and 65.7% would accept the RSV vaccine in future pregnancies. A counseling point for clinicians is stronger emphasis on the ramifications for the child, because this may lead to higher acceptance of the vaccine. Additionally, participants reported the desire for more time to receive the vaccine, suggesting clinicians should make patients aware of it before 32 weeks of gestation.

An unexpected finding included 46.1% of eligible individuals not being offered the RSV vaccine. There was a significant increase in vaccine offering over the months, presumably as the vaccine was worked into clinician workflow. This offers an area for future study regarding improved implementation strategies.^6^ Our data demonstrate that RSV vaccine hesitancy has many potential sources. Understanding patient perceptions will be useful to create vaccination strategies in the prevention and control of RSV in the future.

## References

[1] American College of Obstetricians and Gynecologists. Maternal respiratory syncytial virus vaccination. Accessed January 30, 2024. https://www.acog.org/clinical/clinical-guidance/practice-advisory/articles/2023/09/maternal-respiratory-syncytial-virus-vaccination

[2] Centers for Disease Control and Prevention. Respiratory syncytial virus (RSV) infection. Immunizations to protect infants. Accessed January 30, 2024. https://www.cdc.gov/vaccines/vpd/rsv/public/pregnancy.html

[3] Hammitt LL Dagan R Yuan Y Baca Cots M Bosheva M Madhi SA . Nirsevimab for prevention of RSV in healthy late-preterm and term infants. N Engl J Med 2022;386:837–46. doi: 10.1056/NEJMoa2110275 35235726

[4] Kampmann B Madhi SA Munjal I Simões EAF Pahud BA Llapur C Bivalent prefusion F vaccine in pregnancy to prevent RSV illness in infants. N Engl J Med 2023;388:1451–64. doi: 10.1056/NEJMoa2216480 37018474

[5] Mitchell SL Schulkin J Power ML. Vaccine hesitancy in pregnant women: a narrative review. Vaccine 2023;41:4220–7. doi: 10.1016/j.vaccine.2023.05.047 37291023

[6] Morales KF Menning L Lambach P. The faces of influenza vaccine recommendation: a Literature review of the determinants and barriers to health providers' recommendation of influenza vaccine in pregnancy. Vaccine 2020;38:4805–15. doi: 10.1016/j.vaccine.2020.04.033 32499068 PMC7306152

